# Rare Case of an Epithelial Cyst in an Intrapancreatic Accessory Spleen Treated by Robot-Assisted Spleen Preserving Distal Pancreatectomy

**DOI:** 10.1155/2016/9475897

**Published:** 2016-10-25

**Authors:** Willemijn P. M. van Dijck, Vincent P. Groot, Lodewijk A. A. Brosens, Jeroen Hagendoorn, Inne H. M. Borel Rinkes, Maarten S. van Leeuwen, I. Quintus Molenaar

**Affiliations:** ^1^Department of Surgery, UMC Utrecht Cancer Center, University Medical Center Utrecht, Utrecht, Netherlands; ^2^Department of Pathology, UMC Utrecht Cancer Center, University Medical Center Utrecht, Utrecht, Netherlands; ^3^Department of Radiology, UMC Utrecht Cancer Center, University Medical Center Utrecht, Utrecht, Netherlands

## Abstract

Epithelial cyst in an intrapancreatic accessory spleen (ECIPAS) is exceedingly rare with only 57 cases reported since the first publication in 1980. Comprehensive clinical and diagnostic features remain to be clarified. We present a case of ECIPAS in a 21-year-old Philippine woman who was admitted with right upper quadrant abdominal pain. A cystic lesion in the pancreatic tail was discovered and evaluated by computed tomography and magnetic resonance images. Based on clinical and radiological features a solid pseudopapillary neoplasm was suspected. The patient underwent robot-assisted spleen preserving distal pancreatectomy. Pathological evaluation revealed a 26 mm intrapancreatic accessory spleen with a 16 mm cyst, lined by multilayered epithelium in the tail of the pancreas. The postoperative course was uneventful. Differentiating ECIPAS from (pre)malignant cystic pancreatic neoplasms based on clinical and radiological features remains difficult. When typical radiological signs can be combined with scintigraphy using Technetium-99m labelled colloid or Technetium-99m labelled erythrocytes, which can identify the solid component of the lesion as splenic tissue, it should be possible to make the right diagnosis noninvasively. When pancreatectomy is inevitable due to symptoms or patient preference, minimally invasive laparoscopic or robot-assisted spleen preserving distal pancreatectomy should be considered.

## 1. Introduction

Accessory spleens exist in up to 10% of the general population, with a fifth arising within the pancreatic tail [1]. However, presence of an epithelial cyst in an intrapancreatic accessory spleen (ECIPAS) is remarkably rare, with only 42 published reports covering 57 cases in the English literature since Davidson et al. reported the first case in 1980 [2–42]. ECIPAS is often misdiagnosed as a cystic neuroendocrine tumor or solid pseudopapillary tumor, resulting in unnecessary surgical resection. Since ECIPAS is a nonneoplastic lesion, surgery is not indicated. Clearly, a correct preoperative diagnosis is a key and can avoid unnecessary surgery. Currently, however, comprehensive clinical and imaging features remain to be clarified.

Here we present the first case of an epithelial cyst in an intrapancreatic accessory spleen treated with robot-assisted minimally invasive spleen preserving distal pancreatectomy, using the da Vinci surgical system. Although standard protocol for cystic lesions of the pancreas was followed, including CT and MRI evaluation and presentation in a multidisciplinary meeting, the correct diagnosis was not reached. Clinical, radiological, and pathological features are discussed to contribute to a better understanding of these rare lesions, thereby aiming at a reduction of performing unnecessary surgery.

## 2. Case Report

**Figure 1:**
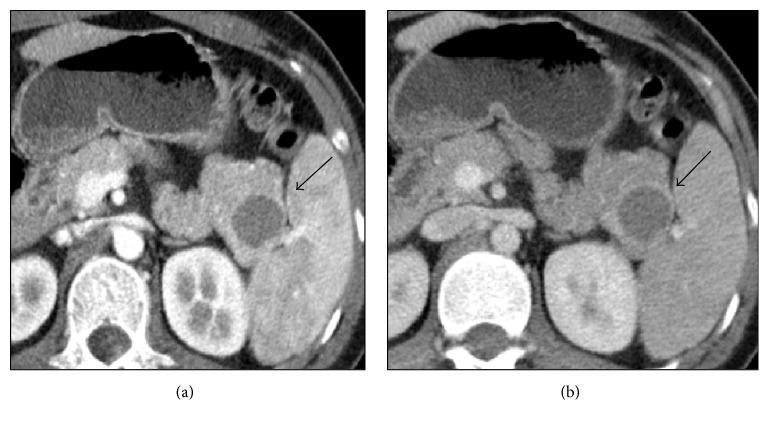
(a) Arterial and (b) portal-venous phase CT, confirming cystic lesion in pancreatic tail without soft-tissue component.

**Figure 2:**
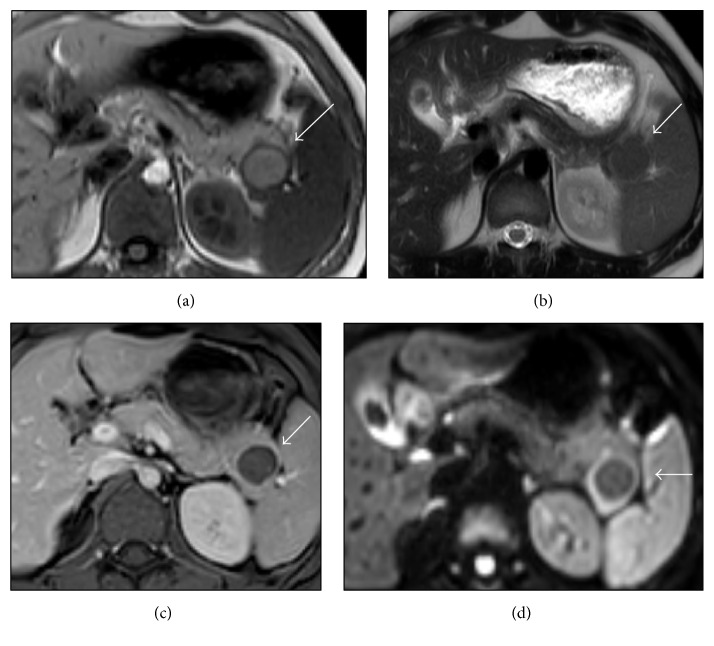
MRI exam showing (a) T1-weighted image, (b) T2-weighted image, (c) Gd-enhanced image, and (d) DWI image of the lesion. Note how DWI best demarcates soft-tissue rim surrounding cystic component, with equivalent signal intensity to spleen.

A 21-year-old Philippine woman was referred to the University Medical Center Utrecht with right upper quadrant abdominal pain since one year. An initial abdominal ultrasound showed cholecystolithiasis. Incidentally, a cystic lesion was detected in the tail of the pancreas. Additional CT imaging exposed a 24 mm cystic lesion in the pancreatic tail, in close proximity to the spleen (Figure 1). The caudal wall of the cyst appeared to be thickened and enhanced. Following our protocol for cystic lesions of the pancreas, a subsequent MRI was performed and revealed a 31 mm cystic lesion with hyperintensity on T1-weighted images, indicative of previous haemorrhage (Figure 2(a)). Enhancement of the cystic wall was seen in the venous phase, with some gadolinium enhancing of the center of the lesion during the arterial phase (Figure 2(c)). The rim of the lesion showed some diffusion restriction on diffusion-weighted imaging (DWI) (Figure 2(d)).

The patient had no medical history and additional physical examination and laboratory tests were normal. The patient case was further discussed in a multidisciplinary meeting and a solid pseudopapillary neoplasm (Hamoudi or Frantz tumor) with past haemorrhage was considered as the most likely diagnosis based on clinical and radiological findings. Owing to the low malignant potential of the suspected solid pseudopapillary neoplasm in the tail of the pancreas, a robot-assisted spleen preserving distal pancreatectomy was advised. In addition, a cystic neuroendocrine tumor and mucinous cystadenoma were in the differential diagnosis. Due to questionable sensitivity, endoscopic ultrasound is not part of standard work-up for pancreatic (cystic) lesions at our institution [43]. Following this, we decided not to perform additional endoscopic ultrasound since the indication for resection of the hypervascular cystic lesion was already established.

**Figure 3:**
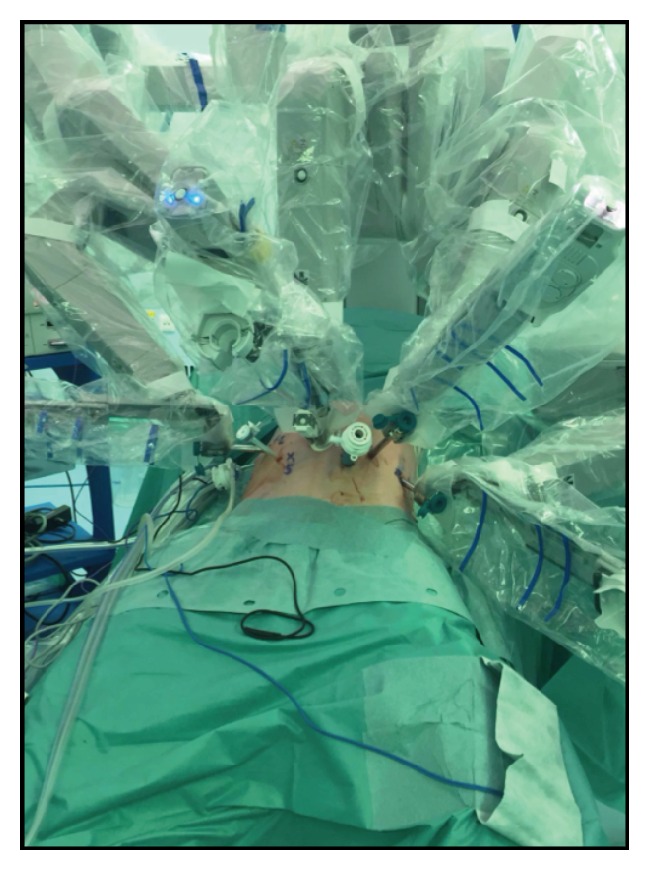
Port positioning for robot-assisted spleen preserving distal pancreatectomy.

Robot-assisted minimally invasive spleen preserving distal pancreatectomy was performed using the da Vinci system (Intuitive Surgical Inc, Sunnyvale, CA) (Figure 3). The patient, under general anaesthesia, was placed in lithotomy position tilted to the left side. Pneumoperitoneum was established and the camera was placed in the right pararectal line. First, the gastrocolic ligament was divided while preserving the gastroepiploic artery. Mobilisation began at the neck of the pancreas in order to achieve control of the proximal splenic artery. The duodenum was exposed, and the superior mesenteric vein was identified at the root of the mesentery. Both the splenic artery and the vein were isolated and left intact. Taking sufficient margin, the pancreatic tail was transected with a stapler device and extracted from the abdominal cavity in an endobag. The total operation time was 2 hours and 4 minutes, with a total blood loss of 20 mL.

**Figure 4:**
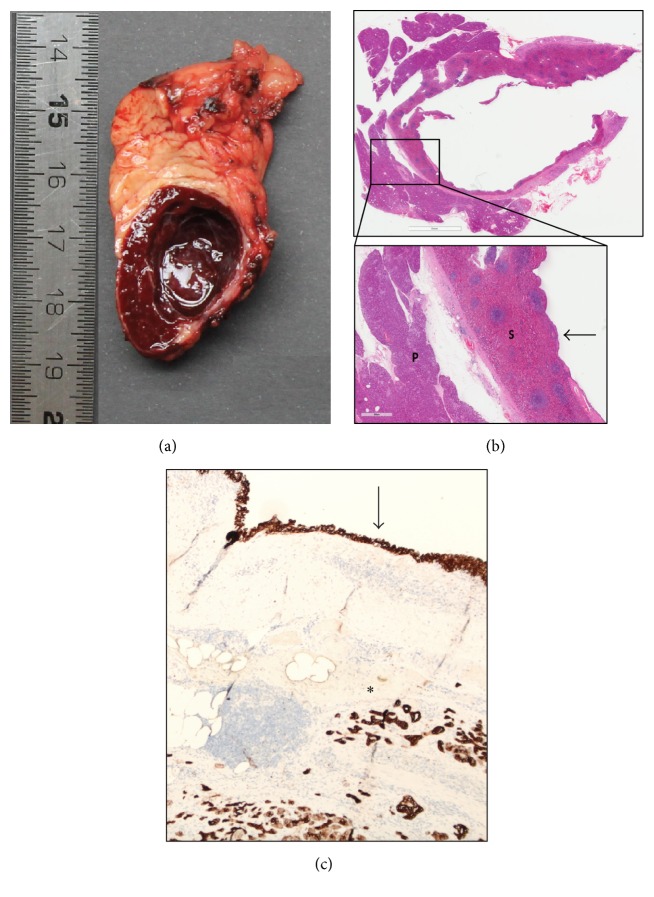
(a) Photograph of the cut gross specimen showing well-defined splenic tissue with a unilocular cyst. (b) Hematoxylin-eosin staining showing pancreatic parenchyma (P) and adjacent splenic parenchyma (S) and the cyst wall lined by multilayered epithelium without atypia (arrow). (c) Cytokeratin 5 staining showing positivity in the cyst lining (arrow) and in surrounding pancreatic parenchyma (asterisk) indicating epithelial lining.

Macroscopically, the specimen showed a well-demarcated and encapsulated dark-red lesion with a diameter of 26 mm harbouring a 16 mm cyst (Figure 4(a)). Microscopically, the lesion consisted of normal splenic tissue with alternating red and white pulp surrounded by a thin fibrous capsule (Figure 4(b)). A cystic space lined by stratified squamous epithelium without atypia was present within the splenic tissue. Immunohistochemical staining demonstrated that the epithelial lining was positive for Cytokeratin AE1/AE3 and Cytokeratin 5 but negative for Calretinin D2-40 and CD34, showing that this was indeed epithelial lining and not mesothelial lining, a lymphangioma, or a hemangioma (Figure 4(c)). No papillary or pseudopapillary structures were observed. Based on these findings, ECIPAS was established as the final pathological diagnosis. The postoperative course was uneventful and the patient was discharged from hospital after five days.

## 3. Discussion

In a report on 2700 necropsies, approximately 10% was found to have an accessory spleen [1]. These accessory spleens were typically located in the splenic hilum (80%) or the pancreatic tail (17%). An epithelial cyst arising in such an intrapancreatic accessory spleen remains a rare entity. However, the increasing use of abdominal CT-scans and other imaging seems to lead to an increasing number of detected cases [30, 33]. This case report shows that, although protocols with respect to cystic lesions of the pancreas were followed and the patient was evaluated in a multidisciplinary meeting, diagnosing ECIPAS may be quite challenging. Presently, there are no accurate criteria to differentiate preoperatively between a (pre)malignant cystic neoplasm of the pancreas and ECIPAS.

When reviewing our own case and all other documented cases (*n* = 57) [2–42], we found that ECIPAS occurs slightly more frequent in woman than men (32 : 25) and is diagnosed mostly at middle age, with a median age of 45 years (range 12–75 years). Interestingly, the majority of patients (*n* = 44), including our patient, are from Asian descent, suggesting that racial factors and genetic background might play a role in the genesis of this lesion. Most often ECIPAS is an incidental finding as most patients (32 of 57) are asymptomatic. However, some patients reported nonspecific complaints such as abdominal pain, weight loss, nausea, and vomiting. When documented, serum CA 19-9 was elevated in 24 patients and was normal in 18 patients. It is argued that high CA 19-9 originates directly from the squamous epithelial lining and high serum levels are caused by trauma or increased intracystic pressure [8]. The median maximum diameter of the cystic lesions was 34 mm (range 14–150 mm) and all lesions were located in the tail of the pancreas. Cysts were described as multilocular in 26 patients and unilocular in 31 patients. Every patient underwent either open (*n* = 46) or laparoscopic (*n* = 10) distal pancreatectomy. Although improved surgical techniques have resulted in lower morbidity and better cosmetic results, laparoscopic and open distal pancreatectomy still result in mortality rates of 0.4% and 1.2%, respectively [44]. Correct diagnosis would therefore save patients from unnecessary surgery and its associated risks.

The presented patient was the first to undergo robot-assisted minimally invasive spleen preserving distal pancreatectomy for ECIPAS. By performing this procedure robotically, surgeons experience more freedom of movement of the surgical instruments, elimination of tremor, and a three-dimensional vision of the operative field. These advantages lead to improved precision in operation technique and may lead to a higher success rate of spleen preservation when performing distal pancreatectomy. Documented advantages of robot-assisted surgery over laparoscopic and open distal pancreatectomy include reduced blood loss, fewer complications, less postoperative pain, faster recovery, and thus a shorter hospital stay [45]. However, robot-assisted surgery also has some important limitations; robotic arm-interference makes it difficult to operate in multiple quadrants of the abdomen, the operation time is generally longer, and associated costs are higher.

Since 1980, a correct preoperative diagnosis of ECIPAS has been reported in only five cases [15, 21, 26, 32]. These accurate diagnoses were partly based on the smoothness of the cystic inner wall and solid component, a differentiating morphological feature suggestive of a benign tumor [26]. Additionally, epithelial cysts commonly show low signal intensity on T1-weighted images and high signal on T2-weighted images [21]. Lastly and most importantly, the density of tumor's solid component was recognized to match splenic tissue on postcontrast CT and MRI. On MRI, the ectopic splenic tissue shows a spleen-like signal on DWI (See Figure 2(d)). The presence of a relatively large amount of splenic tissue surrounding the cyst could thus make a correct diagnosis more likely with careful examination. However, if the amount of splenic tissue is small, accurate diagnosis before surgery remains challenging. When the presence of intrapancreatic splenic tissue is suggested on initial imaging, several techniques could be used to identify the ectopic splenic tissue, including Technetium-99m sulfur colloid scintigraphy and Technetium-99m heat-damaged red blood cell scintigraphy [46]. Splenic tissue traps up to 90% of the injected blood cells. When subsequent single-photon emission computed tomography (SPECT) imaging shows focal uptake in the pancreatic tail, a finding that is consistent with intrapancreatic splenic tissue, the correct diagnosis of an intrapancreatic spleen could be feasible [47]. However, compared to the normal spleen, uptake will be lower in an intrapancreatic spleen as it contains only a small amount of functioning splenic tissue. As yet, no malignant development of ECIPAS has been reported. When this benign lesion is correctly diagnosed, the patient can be discharged from follow-up.

In conclusion, ECIPAS is a rare entity with undefined criteria for preoperative diagnosis. However, we propose that, especially in middle-aged Asian patients, ECIPAS should be included in the differential diagnosis when considering cystic lesions of the pancreatic tail. In case of radiological signs of a smooth cystic wall, homogenous enhancement, and matching density of the solid component of the cyst and the adjacent spleen, the diagnosis of ECIPAS should be taken into consideration and Technetium-99m scintigraphy or SPECT-imaging should be performed. Further understanding of clinical and imaging features might lead to better diagnoses and might prevent unnecessary surgery for this benign lesion. When pancreatectomy is inevitable due to symptoms or patient preference, minimally invasive laparoscopic or robot-assisted spleen preserving distal pancreatectomy should be considered.
